# GAKTpore: Stereological Characterisation Methods for Porous Foams in Biomedical Applications

**DOI:** 10.3390/ma14051269

**Published:** 2021-03-07

**Authors:** Gareth Sheppard, Karl Tassenberg, Bogdan Nenchev, Joel Strickland, Ramy Mesalam, Jennifer Shepherd, Hugo Williams

**Affiliations:** 1 School of Engineering, University of Leicester, Leicester LE1 7RH, UK; kt199@le.ac.uk (K.T.); bn55@le.ac.uk (B.N.); jcjs2@le.ac.uk (J.S.); js1005@le.ac.uk (J.S.); hugo.williams@le.ac.uk (H.W.); 2 School of Physics and Astronomy, University of Leicester, Leicester LE1 7RH, UK; rm558@le.ac.uk

**Keywords:** pore analysis, homogeneity, scaffold, metal foams, space holders, porous materials, tissue engineering, 2D biomaterials

## Abstract

In tissue engineering, scaffolds are a key component that possess a highly elaborate pore structure. Careful characterisation of such porous structures enables the prediction of a variety of large-scale biological responses. In this work, a rapid, efficient, and accurate methodology for 2D bulk porous structure analysis is proposed. The algorithm, “GAKTpore”, creates a morphology map allowing quantification and visualisation of spatial feature variation. The software achieves 99.6% and 99.1% mean accuracy for pore diameter and shape factor identification, respectively. There are two main algorithm novelties within this work: (1) feature-dependant homogeneity map; (2) a new waviness function providing insights into the convexity/concavity of pores, important for understanding the influence on cell adhesion and proliferation. The algorithm is applied to foam structures, providing a full characterisation of a 10 mm diameter SEM micrograph (14,784 × 14,915 px) with 190,249 pores in ~9 min and has elucidated new insights into collagen scaffold formation by relating microstructural formation to the bulk formation environment. This novel porosity characterisation algorithm demonstrates its versatility, where accuracy, repeatability, and time are paramount. Thus, GAKTpore offers enormous potential to optimise and enhance scaffolds within tissue engineering.

## 1. Introduction

The ultimate goal in tissue engineering is to replicate native environments, allowing optimised regeneration of tissues. Difficulties arise due to the heterogeneous structure of tissues, where each tissue has its own three-dimensional extra-cellular matrix (ECM) organisation. Significant evidence suggests that cells within a tissue engineering scaffold, align their ECM to the scaffold structure [1,2,3,4]. Several chemical and biological phenomena, including nutrient diffusion and migration of cells are heavily dependent on pore size and connectivity [4]. Therefore, significant effort is spent on design optimisation of tissue engineered scaffolds, but without thorough characterisation of produced structures, it is impossible to correlate structure with cellular response [5]. Therefore, pore size, shape, and heterogeneity are key parameters for consideration. An extensive variety of materials have been considered for tissue engineering, taking into account not only chemical and biological response but also the mechanical and structural performance of the tissue they intend to repair or replace. [6,7].

Metal foams are a modern class of low density porous materials whose mechanical, electric and thermal properties, provided by their base metal constituents, enable a variety of useful functional applications [8]. Currently, they are used for biomedical applications [9], filtration methods [10,11,12], heat exchangers [13], fuel cell systems [14], lightweight structures [15], energy absorption [16], and sound control system environments [17]. Their diverse material properties stem from a variety of production methods, allowing precise microstructural tailoring to suit the desired application. Depending on the manufacturing route, a metal foam can possess either an open or closed porous structure, where open porosity is a measure of pore connectivity within the structure and closed is a measure of the free space in the body. Open porous metallic structures offer significant potential for the regeneration of load bearing bone structures, not just due to the accessible pore structure for cells and nutrient diffusion, but also as a consequence of their reduced effective modulus compared to the solid material. Biomechanical optimisation for bone repair requires a material with sufficient strength and toughness to undergo physiological loading, coupled with a stiffness low enough to not shield the surrounding bone from loading and causing bone resorption [18].

To increase osseointegration in implants, optimisation is required through careful characterisation of the microstructure in combination with process variable tailoring. It has been shown that the utilisation of prolate or elongated pores can be applied in order to mimic the anisotropy of native bone mechanics [19]. The tailoring of pore volume fraction and distribution promotes cell regeneration and vascularisation [20]. Whilst porosity of the order 100–200 μm is more commonly the range reported for optimised osseointegration [21,22], there is significant interest in the role of microporosity (<10 μm). The high surface area resulting from this fine scale porosity has a considerable effect on protein adsorption, with the capillary forces generated by the microporosity improving the attachment of bone related cells on the scaffold surface [23]. The utilisation of promising new microporous surface coatings, demonstrate strongly interwoven bone trabeculae between pore struts, increasing adhesion between implants and tissue. Exploiting the high surface to volume ratios with microporosity, creates the long term stability of implants [24].

The metal foam production methodology impacts the final pore morphology, where some processes such as the sacrificial space-holder method result in irregularly shaped pores [25]. The sacrificial space holder method utilises the concept of mixing two powders together, with one later being extracted to create a controlled porosity and pore size. The resultant pore size, range, shape, and orientation have a strong correlation with mechanical properties [26]. In addition, the pore dispersion, mean pore size and range are largely influenced by the processing route and agglomeration behaviour of the material. Pore range has been correlated with the permeability and flow rate material properties, where a larger pore size distribution increases permeability for the same mean pore sizes [27]. Compared to spherical, prolate pores are shown to reduce compressive strength and negatively influence bulk mechanical properties, such as the stiffness ratio of metal foams [28]. Refining the pore size distributions provides quality assurance of a materials performance for consumers, thus, potentially improving the safety and reliability of products. Within industry, pore size ranges are set by the manufactures in terms of required material properties and available powder size distributions.

When it comes to the optimisation of scaffold structures for soft tissue repair, the stiffness of metallic foam far exceeds that of the native tissue and consequently would prove deleterious to cell response [29]. Collagen represents the major structural protein of human tissue and it has been applied as the material of choice for a wide range of three-dimensional scaffold structure for tissue regeneration [1,30,31]. Lyophilisation or freeze-drying is a flexible technique able to produce relatively homogeneous, highly interconnected scaffold structures [32,33]. What the technique also offers is flexibility; through careful control of processing parameters such as freezing time and sublimation temperature, a range of pore structures can be produced [34,35]. With the focus on generation of very controlled, reproducible pore structures there is an obvious desire to be able to fully characterise all structural variables. However, analysis of fibrous structures is no trivial task due to the significantly reduced contrast between scaffold and pore features.

In the case of open porous foams, porosity analysis usually occurs via simple pressure drop readings, or gas absorption methods, neither allow the morphology to be fully characterised [36]. Furthermore, these methods provide no measure of the degree of closed porosity. Pore morphology characterisation is typically limited to mean particles size and porosity, determined by manual measurement of scanning electron microscope (SEM) images using basic functions, such as the line tool in the widely used freeware, image processing package, ImageJ (NIH, MD, USA). However, simple porosity and pore area measurements are insufficient to fully quantify the diversity of pore morphologies observed within foams and scaffold structures, thus, limiting their potential performance.

The great advances in computational power in recent years have facilitated the development of a variety of bulk microstructural feature extraction and characterisation digital tools [37,38]. Their main purpose is to bridge the link between materials characterisation and computation to improve the understanding of process-property relationships and to facilitate the development of sophisticated defect modelling approaches, and eventually, Artificial Intelligence. With these new advances in computation power and new data extraction techniques, the surface morphology of materials can be quickly digitalised, allowing rapid and accurate acquisition of statistical data [39].

Typically, open source 2D image processing software is developed for studying porous structures in geological and biomedical sciences [40,41], such as JPor, BoneJ, and DiameterJ [39,41,42] available in ImageJ. However, most user-designed plugins are limited in their application and validation, and only provide basic pore morphology analysis (porosity measurements). Commercial software packages such as AVIZO, PoroMetric, GeoDict offer a wider range of measurements, but are costly and restrictive, stifling the open-source collaborative working environment. Typically, performing 2D particle distribution measurements require supplementary software or plugins such as BioVoxxel Toolbox (ImageJ), which quantify homogeneity/inhomogeneity using nearest neighbour relationships based on centroid measurements, assuming near circular pore shapes. However, such methods do not account for the complexities and irregularities of pore geometries, hence are not viable for quantifying homogeneity (pore dispersion) for highly irregular porous materials. As it stands, no software for comprehensive investigation of metal foams exists, despite their key application in the biomedical area. Consequently, there is a strong requirement for a combined open-source porosity and homogeneity quantification software, designed and validated for a wide range of morphology characterisation tools.

In this work the aforementioned challenges are addressed by developing a novel algorithm for bulk porous foam characterisation and material optimisation: GAKTpore. This new software is designed for investigation and quantification of pore morphology differences in foam structures, fabricated via differing methodologies within the field of tissue engineering and more broadly. The proposed algorithm offers a wide range of material-structure applicability and is rigorously validated across multiple length scales, morphologies, and features. Further, GAKTpore offers new metrics for quantifying pore dispersion/homogeneity as well as measures for the proportion of convex/concave curvature of features, which in recent studies has been shown to significantly influence cell adhesion and proliferation [43,44,45,46]. In this study, fibrous structures, such as metallic foams produced through the space-holder route and collagen manufactured through lyophilisation, are investigated with GAKTpore revealing new relationships between homogeneity and processing route.

In the following study, the framework of the new open-source algorithm: GAKTpore, is reported, Section 2. Metrics such as porosity, local area fraction (pore dispersion/homogeneity), shape factor (circularity, waviness, and aspect ratio), pore size, and largest sphere fitting through a given pore (LSTP), are all introduced. Synthetic image generation as well as biomaterial fabrication and imaging, utilised for validation and characterisation, respectively, are outlined in Section 3. Collagen scaffolds and porous copper foams (utilised as a surrogate for titanium) are characterised and reported in Section 4. All validation data is also included demonstrating the accuracy and broad range of the algorithm applications. In Section 5, porosity of scaffolds and foams are characterised and discussed within the context of manufacturability and standardisation for biomedical applications and tissue engineering. Final conclusions and future applications are drawn in Section 6.

## 2. GAKTpore Algorithm and Metrics

**Figure 1:**
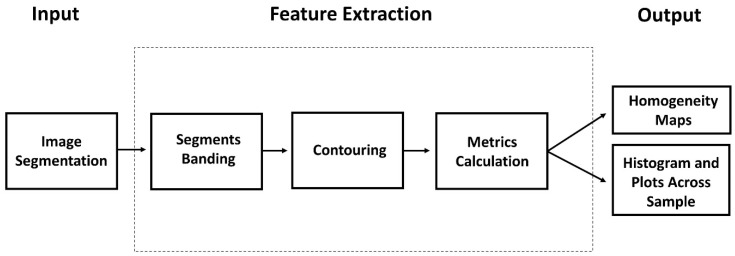
Flow diagram of the GAKTpore algorithm. An input micrograph is pre-processed via image segmentation methods then divided into bands. Pores or features are then extracted through contouring and processed through FFT algorithm. Once obtained multiple metrics such as porosity, local area fraction (pore dispersion/homogeneity), shape factor (circularity, waviness and aspect ratio), pore size and largest sphere fitting through a given pore (LSTP) are calculated. At the final stage, homogeneity maps are generated and results are saved.

GAKTpore is designed as a rapid, accurate, and comparable pore morphology analysis characterisation tool. The software can run on any platform with a Python add-on installed. In this work, SEM micrographs 1552 by 1051 pixels are analysed on a HP EliteOne 800 G2 23 (Windows 10, 8GB of RAM, Intel i5-6500). The total processing time for each image is between 8–10 s per micrograph. Large sample maps consisting of multiple stitched SEM micrographs, 14,784 by 14,915 pixels were also analysed with a total processing time of 9 min per map. The algorithm processing path is illustrated in Figure 1. The GAKTpore algorithm incorporates a new FFT (Fast Fourier Transform) pore contouring technique for improved accuracy and novel metrics for gaining novel insights into pore morphologies. In the following sections, each stage of the GAKTpore algorithm is explained in detail.

### 2.1. Image Segmentation

**Figure 2:**
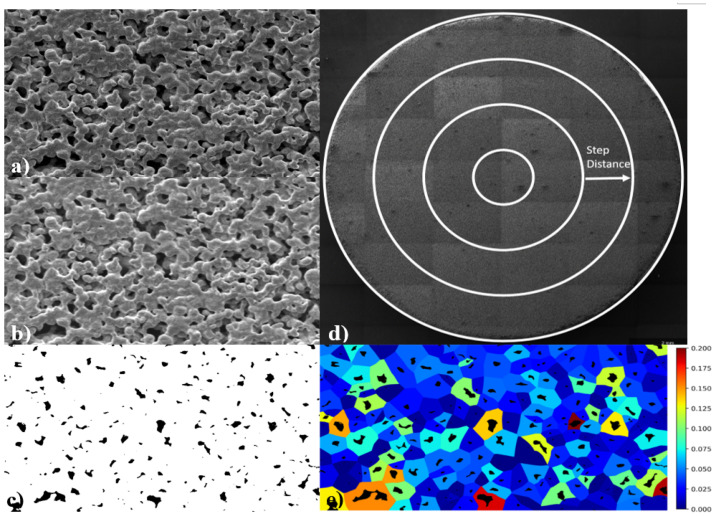
SEM micrograph of image changing through segmentation and thresholding process (**a**) Original image, (**b**) Image after FFT, (**c**) The binarised image after a threshold value has been selected, (**d**) Demonstration of how the image is divided up into ringed segments set by the user defining the step distance (**e**), local pore area fraction map displaying homogeneity of the material.

Image segmentation is required to visibly identify all pores from the solid. This is achieved by, firstly, converting the image to grayscale, as shown in Figure 2a. Then, an FFT band pass filter (FFTBP) is applied to increase image definition between the porous and solid areas (Figure 2b) and remove noise, significantly improving the accuracy of image thresholding. An FFTBP filter removes low and high frequency features, by the application of a strong Gaussian in the Fourier space. This method provides an even brightness throughout the image and removes any noise effects caused by the imaging equipment.

It is pertinent to note, that a reduction in pixel density reduces the accuracy of the algorithm. Through vigorous testing, it is recommended that the length of the features of interest are at least 12–15 pixels, with an increase in pixel density more accurate the representation of the extracted features. A minimum mean feature size to step length ratio should be implemented of 1:10 to allow a large enough sample area and frequency of readings to be selected, where features divided between ringed segments have negligible effect on end mean result.

Before algorithm application, the image is binarised according to a threshold and a scale value. To keep the versatility of the algorithm for various application’s this step is executed in ImageJ [47]. The image/micrograph is manually thresholded until a satisfactory value is determined, as shown in Figure 2c. The image scale is obtained from the scale bar, with both values entered into the algorithm.

### 2.2. Contour Determination, Porosity and Pore Area

**Figure 3:**
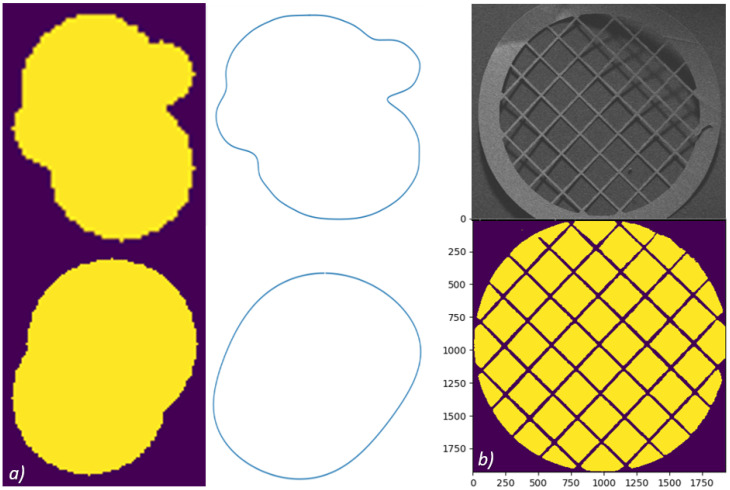
(**a**) Purple background are the produced synthetic images and white background are the contours detected by the algorithm from the synthetic images (**b**) 75 mesh Athene TEM copper grid, the top image is the SEM micrograph and the bottom are the plotted contours detected by GAKTpore.

Pore contouring is determined by applying an already validated perimeter function, part of OpenCV [48]. The function yields a single perimeter of all contours within the image (Figure 3a). Due to the possibility of getting a pore within a pore, only the external contours are considered. Total porosity is calculated as an area fraction by summing all pore pixels in the examined area and dividing by the total image area. Assuming the pore is a perfect circle, the calculated contour areas (pore areas) are used to find the circular pore diameter.

### 2.3. Segment Banding

To visualise feature variation across a sample, functions such as mean pore size and porosity can be plotted as a function of distance. The image is split into ringed segments starting from the centre and moving outwards. The step distance between the rings is determined by the user. For each segment band, all property data is stored for each calculated contour in the image (Figure 2d).

### 2.4. Pore Dispersion/Homogeneity Determination

In this section, a novel methodology for mapping feature homogeneity is proposed. Algorithms in the literature generally rely on number density variation techniques, Nearest-Neighbours, Voronoi networks and Point clouds to calculate homogeneity [49], however, all of these algorithms are developed for points/centroids but not for complex shapes. The features studied in this work possess complex morphologies that cannot be easily approximated to a point. This leads to large deviations in results when any of the aforementioned algorithms are applied.

A special neighbourhood algorithm is used to relate every point/pixel in the image to the nearest contour. An area of free space around every contour in the image is obtained (territory area). This area is influenced by the internal pore shape, the neighbouring shapes and their proximity. Homogeneity is obtained from the local area fraction ratio, calculated by dividing the pore area over the territory area. Visualisation is facilitated by a colour map which provides a contrast between different area fraction ratios (Figure 2e).

### 2.5. Calculation of Max Radius-Largest Sphere Fitting Through a Pore (LSTP)

In the situation where pores demonstrate highly irregular geometries (as seen in Figure 2c), the perfect circle assumption largely overestimates the pore radius, the LSTP (largest sphere fitting through a pore) method is widely used as a better approximation.

For each contour, a linear interpolation function is used to produce twenty times the number of points to original points to increase the accuracy of the later applied FFT bandpass filter. An FFT low bandpass filter is applied to make the contour shape continuous and smooth (Figure 3a). For the FFT to produce the same contouring effect regardless of size, the low bandpass filter cut-off value is scaled according to the length of straight lines to curved lines ratio in *x*-axis and *y*-axis, where the FFT is applied in the *x*-axis and *y*-axis separately. Straight lines are the hardest to reproduce with continuous signal and require very accurate cut-off value.

To calculate LSTP a memory and computationally efficient method is utilised. A premade point grid is created around each contour (pore), the size of which is determined by the maximum width and height, where only grid points inside the contour are kept. For each point in the grid, the nearest distance to the edge of the contour is calculated with the maximum being the LSTP radius. To improve the accuracy, the above steps are repeated using a finer grid around the point of the found radius. The final resulting length is used to determine the maximum particle size that can fit through the pore.

### 2.6. Calculation of Shape Factors

Circularity is calculated using Equation (1) (ISO9276-6), where contour area is determined using the Shoeslace theorem, with *A*—area of pores and *P*—perimeter:

(1) $$f_{Circularity}=4\pi A/P^{2}$$

Waviness is defined as the ratio between convex parameter to total parameter [50]: where the curve is concave if the centre of curvature is outside of the shape, and vice versa. In other words, waviness is the fraction of the total perimeter that is locally convex:

(2) $$f_{Waviness}=P_{Convex}/P$$

Generally, waviness is challenging to calculate in complicated discrete shapes as the centre of curvature is dependent on the normal vector, hence, on the first and second order derivatives of the contour. Derivatives of discrete shapes produce highly inaccurate results due to their non-linearity. However, due to the developed and applied FFT lowpass filter (converts the discrete shapes to continuous), accurate derivatives and normals become straight forward to obtain.

Aspect ratio is usually expressed as the relationship between the largest diameter and smallest perpendicular to it (ISO9276-6). The minimum diameter of each shape is found by rotating each contour around its centre between −180° and 180°. The longest perpendicular distance to the minimum diameter is taken as the maximum diameter value. The aspect ratio is calculated by both values as follows:

(3) $$A_{R}=d_{min}/d_{max}$$

## 3. Material Fabrication and Imaging

### 3.1. Digital Synthetic Features for Validation

Algorithm validation was achieved by comparing results to pore shapes of known sizes. Firstly, a grid was created, then circles were plotted on the grid using the OpenCV draw circle function. The generated circles were plotted to overlap each other in order to provide the complex pore shapes usually encountered (Figure 3a). The radii of the circles were set by the user during the configuration of the synthetic pores. The largest radius circle that fits the pore is detected by the algorithm. Circles with different pixel radii and circle centre coordinates were plotted to make pore like shapes. Eight different pore configurations were created using this technique. The radii were compared with the measurements recorded from the GAKTpore algorithm and this is reported in the results section, Table 1.

To validate the circularity and aspect ratio shape factors (Section 2.6) a method similar to the synthetic pores was used to generate shapes of known dimensions. The OpenCV circle and polygon fill function were used to create basic circles and polygons. The shapes were then processed by the GAKTpore algorithm; circularity and aspect ratio values are reported in Table 2. The waviness shape factor is problematic to validate as simple polygons are inadequate, since they did not possess concave/convex regions, thus, requiring a new validation method. The waviness was validated by plotting a half ellipse together with another half ellipse within (as seen in Table 3), generating horse shoe shaped geometries. It is assumed that the inner ellipse represents the concave proportion of the diameter and the outer representing the convex part. The perimeters of the ellipses were numerically calculated using the binomial infinite series and compared with GAKTpore outputted results.

### 3.2. Fabrication of Foams and Scaffolds

A 5 μm, 99.8% spherical copper powder (Titanium surrogate), and 10 μm spherical PMMA powder from Goodfellow (Huntingdon, UK) was weighed and mixed to provide a 50:50 volume fraction. These powders were subsequently cold pressed at 300 MPa and then sintered in an inert argon gas furnace at 760 °C for 1 h

The lyophilised scaffold produced from type 1 insoluble collagen (bovine) produced for the application of a cell filtration during ex vivo platelet generation experiment. These scaffold structures were manufactured through a multi-stage lyophilsation process in order to create a structurally graduated structure that has been extensively analysed elsewhere in terms of pore size and distribution as well as interconnectivity [51,52].

### 3.3. Imaging

**Figure 4:**
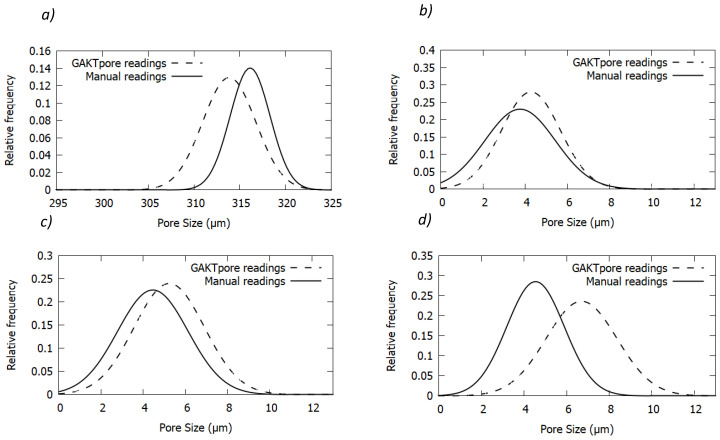
Gaussian fits of histograms comparing data collected from GAKTpore and manual measurements using ImageJ. (**a**) 75 mesh Athene TEM copper grid, (**b**) Platinum sample, (**c**) Copper sample 1, (**d**) Copper sample 2.

**Figure 5:**
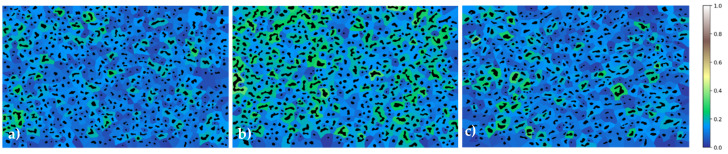
Homogeneity maps plotting local area fraction of pore area to territory area with pores overlaid. Lower ratios (dark blue) correspond to a larger territory area to pore area through a combination of smaller pores and greater pore spacing. (**a**) Copper sample 1, (**b**) Copper sample 2, (**c**) Platinum sample.

For algorithm validation SEM micrographs were acquired of porous copper and porous fibrous platinum foams produced via the space-holder route and deposition route, respectively (Figure 4 and Figure 5). The micrographs were acquired using a FEGSEM (ThermoFisher Scientific, Waltham, MA, USA), FEI Quanta 600, at 200× magnification. A total of 18 SEM micrographs (six for each sample, n = 6) were analysed. A TEM Athene 75 mesh standard copper grid (G210), shown in Figure 3b, with certified dimensions (to be compared with results from the algorithm) was also imaged using the SEM.

**Figure 6:**
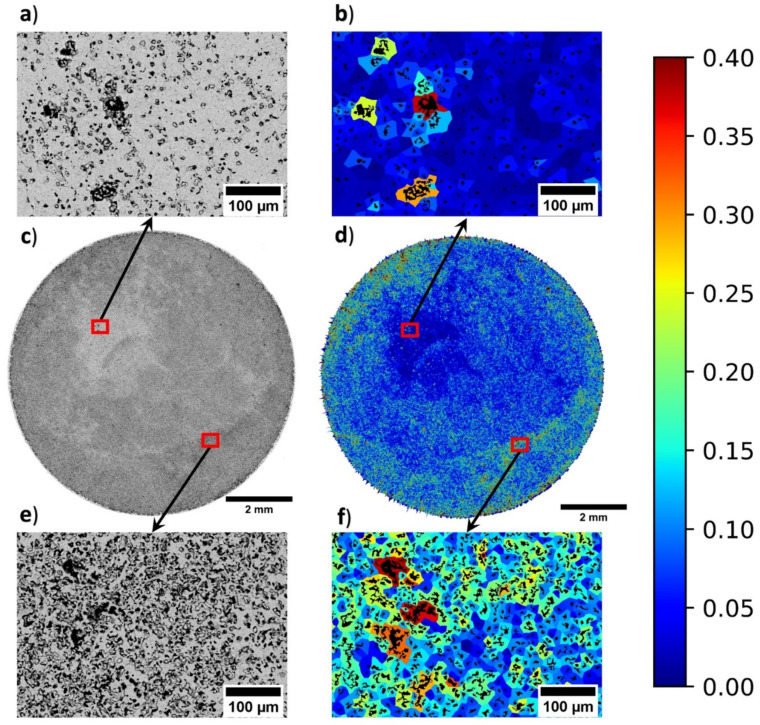
A 10 mm porous copper disk manufactured via the space holder technique, imaged via SEM and analysed with GAKTpore where the colourmap indicates the local area fraction: (**a**) SEM Backscatter micrograph of upper region zoomed, (**b**) Homogeneity micrograph of upper region zoomed, (**c**) Bulk SEM backscatter map (**d**) Bulk homogeneity map, (**e**) Backscatter micrograph of lower region zoomed (**f**) Homogeneity micrograph of lower region zoomed.

For tissue engineering applications a porous copper disk manufactured via the space holder route, was imaged on a Quanta 650 FEG SEM using FEI MAPS 2.1 software (ThermoFisher Scientific, Waltham, MA, USA), shown in Figure 6. The images were taken using a back-scatter detector to provide the best contrast between the base material and the pores. The individual micrograph tiles were recorded using a HFW of 2.072 mm, dwell time of 20 μs, and achieved a resolution of 3072 × 2048 per tile. Image thresholding was determined using ImageJ and the bulk stitched SEM maps analysed using the GAKTpore algorithm.

**Figure 7:**
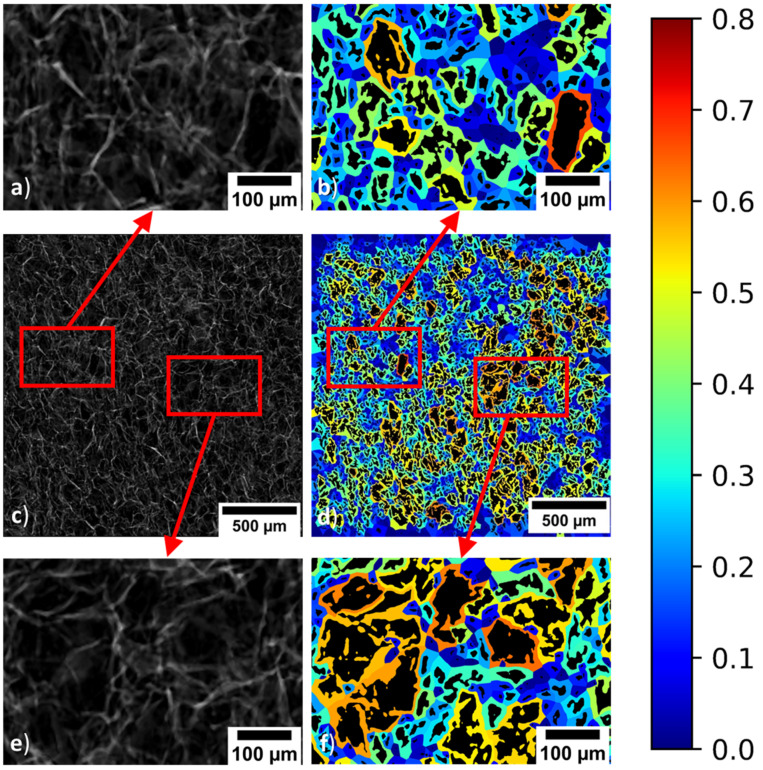
A collagen scaffold imaged using micro CT and analysed with GAKTpore where the colourmap indicates the local area fraction: (**a**) Micro CT micrograph of upper region zoomed, (**b**) Homogeneity micrograph of upper region zoomed, (**c**) Micro CT map (**d**) Bulk homogeneity map, (**e**) Micro CT micrograph of lower region zoomed (**f**) Homogeneity micrograph of lower region zoomed.

To further demonstrate the applicability of the method both in terms of scaffold and imaging source, the lyphilised scaffold was imaged using a Skyscan 1272 Micro-CT system (Bruker, Kontich, Belgium). Punched samples with a 5 mm diameter were scanned with a pixel size of 3 micron and at an operating voltage of 25 kV. Thirty-six slices were combined into a single image using ImageJ and then upscaled by a factor of 25 using a bicubic interpolation, shown in (Figure 7). The combined collagen scaffold slice threshold was determined using ImageJ and analysed using the GAKTpore algorithm.

### 3.4. Manual Measurement

In order to validate GAKTpore, the standard self-measuring method was used. The smallest diameter of the pore was measured manually from the SEM micrographs using the ImageJ line tool. Fifty independent measurements at random locations on six micrographs were acquired on three different samples. In total, one hundred and fifty readings for each sample; two samples of porous copper made via the space holder method and one sample of porous fibrous platinum were made via the deposition route. For each sample, three hundred data points are recorded and compared against the algorithm results. Manual measurements of the Athene copper grid were also taken using the line tool in ImageJ. Twelve measurements were taken of the grid diameters and then compared against the diameters calculated in GAKTpore.

## 4. Results

### 4.1. GAKTpore Validation on Synthetic Images

#### 4.1.1. Validation of LSTP

The GAKTpore analysis was performed on 8 synthetically created pores. Table 1, below compares the largest radii from each pore and the GAKTpore radii calculated from the image. The accuracy is defined simply as the percentage error in the parameter measured using GAKTpore compared to the synthetic baseline value.

The GAKTpore algorithm demonstrates an excellent level of confidence with a mean accuracy of 99.6% for 8 synthetic images. GAKTpore slightly overestimated the radius when there are many similarly sizes radii in the pore configuration. However, the actual overestimation is negligible and did not have a significant effect on the results. In Figure 3a, the purple background images are synthetic, and white are the image contours calculated by the algorithm. The contours have a good similarity with the original synthetic image. The contour is detailed enough to enable a full extraction of all the relevant detail out of the synthetic image with a high level of accuracy and repeatability. From the preceding validation, good confidence in the results is provided by the GAKTpore analysis.

#### 4.1.2. Validation of Shape Factors

The validation of the circularity and aspect ratio is carried out on generated synthetic shapes of calculated circularity and aspect ratio, shown in Table 2. The mean accuracy is 99.5% for circularity and 99.9% for the aspect ratio. The accuracy is defined simply as the percentage error in the parameter measured using GAKTpore compared to the numerically calculated value using the preceding equations. The mean accuracy of each parameter can be defined by the mean of all accuracy values for that parameter.

The validation of the waviness is carried out on the synthetic images in Table 3, where the mean accuracy is found to be 99.13%. Slightly higher errors are found for the smallest inner diameter where the accuracy is 97.8%. For the synthetic shape with inner radius 10 and 15, a small part of the concave curve is selected as convex inducing a small error.

### 4.2. GAKTpore Validation on SEM Micrographs

#### 4.2.1. TEM Athene 75 Mesh Standard Copper Grid (G210)

The Athene copper grid is highly characterised and is chosen as a validation image, dimensions supplied are for approximate hole size of 300 × 300 μm^2^. The mean diameter of manual measurements recorded using the ImageJ line tool and GAKTpore were 315 µm and a mean diameter of 307 µm, respectively; GAKTpore achieving 97.2% accuracy.

Gaussians of the histograms from the copper grid are plotted for the manual and GAKTpore diameter measurements in Figure 4a. They are plotted as function of relative frequency so both sets of readings can be accurately compared and demonstrate a good agreement with each other. It should be noted that the GAKTpore Gaussian is slightly shifted to the left due to the algorithm measuring all the smaller diameters present near to the circumference of the grid.

#### 4.2.2. Micrographs of Sintered Samples

The data collected from each sintered sample (six micrographs) is combined to build up a larger understanding of the characteristics for each sample. Gaussians of histograms are plotted by comparing the manual measurements with the GAKTpore measurements. Table 4 below compares the mean pore sizes (LSTP) calculated from the GAKTpore algorithm and manually recorded readings, where a small deviation is found. The GAKTpore results tend to be slightly higher than those manually measured. In addition, manual results demonstrate a larger pore range. However, all mean values lie within the standard deviations of all plots. The GAKTpore algorithm measures all pores in the image providing a more accurate representation, which can increase the mean pore size.

All Gaussian fits of histograms in this section portray a similar relationship of pore size distribution between GAKTpore and manual measurements. The main difference found is that larger pore sizes are more prevalent in the GAKTpore readings. The sample reading size is much larger for the GAKTpore compared to the manual readings. It is pertinent to note, that due to the frequency in the readings, the smallest pores (lower than 2 µm) do exist in the structure and are detected by the algorithm, but due to their infrequency are not visible on the histogram for the GAKTpore measurements.

#### 4.2.3. GAKTpore Shape Factor and Pore Dispersion Statistics

Many valuable statistics for analysing pore morphology are included in the GAKTpore algorithm. Table 5 contains the mean circularity, mean waviness, mean aspect ratio and mean local area fraction of the micrographs for each sample. It is demonstrated that the porous copper sample 1 pores are the most circular, and fibrous platinum has the least circular pores (as expected of fibrous morphology). Both the copper samples possess a similar mean aspect ratio with the fibrous platinum being lower with more elongated pores. Waviness values are shown to be similar for all samples, therefore, indicating the majority of pores are convex in shape. Using the local area fraction ratio as described in 2.5, the porous copper 2 sample is shown to possess a higher area fraction out of the two copper samples; this is clearly illustrated in Figure 5b.

### 4.3. Tissue Engineering Application

GAKTpore was applied to a 10 mm porous copper disk, as shown in Figure 6. From the results, it is visible that the microstructure does not possess a homogeneous distribution of pores. In the bulk back scattered electron map, the lighter zones correspond to less porosity and a greater area of copper, shown clearly in Figure 6c.

The feature contrast is further accentuated within the homogeneity map (Figure 6d), where a reduction in porosity density corresponds to a lower local area fraction (blue regions). Regions with greater porosity density appear darker on the backscatter SEM map and turquoise/red on the homogeneity map. These regions are more difficult to identify by purely visual examination of the back scattered electron map. However, are clearer when both backscatter and homogeneity maps are viewed simultaneously. In addition to the novel homogeneity maps, the GAKTpore algorithm produces a variety of useful statistics for each microstructure analysed, as illustrated in Table 6.

Figure 7 demonstrates the application of the homogeneity map tool within the GAKTpore software applied to a collagen scaffold imaged using micro-CT. The homogeneity map improves the contrast within the image so that variations in pore size and shape are easily visualised. This is especially useful within micro-CT slices, where the resolution is typically not as high as SEM imaging (Figure 6).

## 5. Discussion

### 5.1. Algorithm

To validate the accuracy of GAKTpore, the software was applied to several different test cases (Figure 3, Table 1, Table 2, Table 3, Table 4 and Table 5). First, the algorithm was tested on synthetic images as their dimensions can be set manually, enabling a simple yet robust method for calculating accuracy and repeatability (Table 2, Table 3). An accuracy between the two methods is determined as 99.6% for LSTP, 99.5% for Circularity, 99.13% for Waviness and 99.9% for Aspect Ratio. The local area fraction was not needed to be specifically validated with synthetic images as it uses the already validated FFT contouring technique (Figure 3a). Furthermore, the LSTP is validated against a 75-mesh copper Athene TEM grid with known dimensions and an accuracy of 97.1% is determined. Consequently, GAKTpore determines four pore morphology characteristics with excellent accuracy. For further verification/industrial application porous copper and platinum foams are analysed using the algorithm and compared with manually recorded measurements. For all but one of the test cases, the difference between manually calculated measurements and GAKTpore is significant (Figure 4). Thus, the industrial method of manually determining pore characteristics is not sufficient for determining the true range of pore morphology features. Consequently, application of GAKTpore can improve the quality assurance of manufactured components.

It is established that the largest source of error is due to bad image segmentation and manual thresholding. Auto-thresholding is not implemented on GAKTpore, since it is highly dependent on the number of materials, the application, and image (micrograph) type and quality. For inclusion of multiple auto-thresholding methods, a GUI is required. Currently, determining the thresholding values for image segmentation first, is achieved manually using ImageJ features and plugins or custom scripts. Other errors associated with GAKTpore occur due to application of the FFT contouring algorithm. The relatively larger errors (~3%) are found to be associated with completely straight lines in shapes such as squares for functions like waviness. Consequently, the algorithm is less suitable for analysing shapes constructed of long straight regions and is more suitable for analysing tortuous features. Further disparities between GAKTpore and manual measurements occurred as a result of measuring the largest diameter of a pore by eye.

From investigating other open source porosity software, it is found that the only similar non-commercial software package available is ImageJ. There are two comparable functions in ImageJ to GAKTpore, circularity and aspect ratio. These are tested in ImageJ using the synthetic images used to validate the functions in GAKTpore. For circularity the accuracy in ImageJ for an equilateral triangle and hexagon is 91.2%, a rectangle and square is 99.5% and a circle is 90%. The aspect ratios in ImageJ showed an aspect ratio of 1 for all the shapes tested, this differs from the GAKTpore algorithm due to a different definition of aspect ratio. The GAKTpore algorithm applies the standard aspect ratio determination method as defined by the International Organisation for Standardisation-ISO 9276-6:2008. Circularity calculated by the GAKTpore algorithm has a much greater mean accuracy of 99.5% compared to a 94.3% in ImageJ. DiameterJ also has circularity and aspect ratio functions but cannot be compared with GAKTpore as fibres within the image are required for the DiameterJ analysis to function. BoneJ and Jpor are also investigated but they are unable to quantify the same features as GAKTpore and so cannot be directly compared. Many plugins were available in open-source software for the calculation of nearest neighbour distributions for pore dispersion. However, none applied a method whereby the contour/edge of the shape was used to determine the polygon/cell around each pore. Therefore, this new method allows the ratio of pore area to localised surrounding territory area, to be evaluated.

The GAKTpore algorithm creates a morphology map that allows the user to visualise how all functions vary across the sample (Figure 2d). The flexibility in the algorithm allows the user to set their own step length to observe how a property varies across a sample, allowing trends in visualisation over different length scales to be observed. This is powerful, as it enables the user to understand how trends, such as mean pore size varies, across the sample surface, which could be affected by different processing parameters.

The measurement of pore homogeneity/pore dispersion uses the pore contour to calculate local interaction with neighbours. This enables the technique to consider the local pore shape and size, and its influence on pore development. This is opposed to the traditional technique that uses pore centroids to form a Voronoi diagram. Such a method is not valid for most cases, since it is only applicable for proportionally very small and round particles/pores when compared with their local spacing. Accounting for pore size and shape allows for the influence of nearest neighbours on pore development to be quantified properly for the first time. As pore formation is a result of the bulk environment, this new method enables the influence of processing parameters on the resultant microstructure to be quantified in manner that is visually intuitive. Furthermore, porous mechanical properties can be improved by altering the processing parameters to improve the homogeneity of pore dispersion within the material.

The algorithm is written in Python as it is an open source programming language. The program allows users to work with large file sizes with much quicker processing times in comparison to ImageJ due to parallel processing implementation. Macro-scale maps with microscopic detail are now possible using dedicated software to stitch SEM micrographs together, allowing the full mapping of a sample. For efficient feature extraction, detailed maps are required, resulting in large file sizes. Dedicated software for feature extraction of large images is sparse, due to the requirement for greater computing power and parallel computing is not easily implemented into a GUI. Working with large file sizes enables full sample characterisation, hence, providing analysis of trends across a sample with greater statistical significance. Therefore, allowing metal foam property optimisation regarding application. Hence, GAKTpore’s ability to provide a bulk stereological examination of a microstructure enables a more detailed and accurate comparison between process and property relationships.

### 5.2. Applications

As previously introduced, there is significant interest in the role of microporosity (<10 μm) due to the considerable effect on protein adsorption and large capillary forces generated improving attachment of bone related cells on the scaffold surface [23]. In this study, copper powder was used as a surrogate to produce microporous foams due to its relative economical pricing and ease to acquire small powder particulate distributions, with the intensions of investigating the space holder viability at being used to produce microporous foams for potential tissue engineering applications.

The space holder technique is often regarded as an inexpensive and simple method to produce porous metal foams. However, the reproducibility of manufacturing porous parts is still major challenge in field and one of the main reasons the space holder technique has not been widely implemented in industry. Arifvianto el al. observed that the porous structural characteristics of titanium scaffolds manufactured via the space holder route did not significantly deviate from those of the green compacts [53]. Concluding that the final porous structural characteristics could be estimated from the green compacts. Therefore, it can be inferred that the greatest influence on the final microstructure is the preparation of powders before pressing and sintering. Thus, an area of great interest to the authors is characterising the dispersion of the space holder within the powder matrix prior to sintering, allowing refinement and highly reproducible microstructures.

Interestingly, from analysing the SEM maps (Figure 6), it is apparent that there are large regions of heterogeneity across the sample which is interwoven with locations of order. The blue regions in the derived homogeneity map (Figure 6b), show clear regions of lower porosity density. Whereas, in Figure 6f, the homogeneity map demonstrates a larger porosity density with larger pore area, that exhibit a greater degree of agglomeration. Therefore, it can be concluded, that insufficient powder mixing has resulted in the agglomeration of regions denser in copper, thus, the derived bulk processing parameters were insufficient to achieve the desired homogeneous porous structure required for application.

When utilising metal scaffolds manufactured via the space holder route, inhomogeneity of porosity distribution has been observed to result in a lower efficiency of stress distribution within the matrix [54]. Therefore, it is imperative to have a homogeneous pore distribution to reduce stress concentration regions within the bone scaffold, to decrease propensity for premature failure of the implant. In terms of utilising the space holder route for the production of microporous foams, further work needs to be completed to improve mixability of the powder to produce homogeneous structures with refined pore size distributions. GAKTpore offers a new method to analyse the effect of processing parameters on the microstructure and bulk simultaneously. Thus, offering new insights into bulk process-property relationships that may have been previously overlooked by only analysing small regions of larger microstructures.

The GAKTpore algorithm was applied to assess the quality of collagen scaffolds, shown in Figure 7. The structures analysed within this work were designed to have structural graduation through their thickness but were assumed to be homogeneous in the field of view analysed here [51]. As demonstrated within this paper, all samples possessed heterogeneity over the bulk cross-section. This is perhaps not a surprise, as inherent within the process of lyophilisation are differential thermal contractions arising due to variations in heat extraction intensity over the bulk microstructure, thus dictating the collagen formation and the resultant morphology [51]. GAKTpore provides a rapid collagen quality assessment tool and can easily quantify morphological feature variation and sample homogeneity/heterogeneity (Table 6). It follows, GAKTpore could be used to improve the quality and capability of collagen scaffolds used in clinical practice by increasing sample homogeneity, where this is desired.

By optimising the effective modulus with pore sizes, shape, range, and dispersion in order to get the best relationship between surrogate tissue/bone strength, nutrient diffusion and cell accessibility idealised conditions for cell migration can be achieved. Characterising and refining the bulk heterogeneity of tissue/foam scaffolds allows matching with tissue ECM for optimum osseointegration, it also allows the refining of processing parameters for creating new microporous coatings for implants [24].

Analysis of circularity and waviness are of increasing interest within the field of tissue engineering. Geometry of individual pores not only influences density, permeability and the amount of tissue produced in the scaffold, but also the speed and nature of tissue deposition [46]. A number of studies have shown the concave or convex nature of the surface to have a significant influence on cell adhesion and proliferation [43,44,45,46]. Rumpler et al. observed that where a surface was not convex, local tissue growth was proportional to curvature and that cells were able to respond to radii of curvature much larger than the cell itself. It has been hypothesised that the combination of large highly non-convex shapes with smaller (mainly convex) pores fitted in between will promote the anchoring of the scaffold at the early time points, diffusion as growth progresses, but also cell adhesion and tissue proliferation [46]. It is for analysis of this sort of complex structure that the full potential of GAKTpore can really be appreciated.

GAKTpore could be used for light weighting of parts for transport applications, to reduce material costs and increase vehicle efficiency; thus, reducing emissions. By reducing pore range then quality assurance is improved, thus, potentially providing a higher performance material. This can be implemented to improve process-property relationships and provide detailed analysis of how processing parameters effect outputs. The reduction of pore range would also allow better reproducibility of parts and higher accuracy of prediction of flow properties.

Further, the implementation of feature extracted data to AI has become a new popular topic of research, as data sets can be imported linking process-property and morphology-property relationships allowing efficient future optimisation of metal foams for specific applications using the relationships discovered.

## 6. Conclusions

GAKTpore is a new accurate, rapid (10 s) and validated shape feature extraction algorithm for scientists and engineers to efficiently and comprehensively analyse pore morphology and homogeneity on a macroscopic scale. The GAKTpore results are shown to be comparable to manual operator measurements and almost an exact match to the synthetic image results, with >99% accuracy. The speed of this analysis allows straightforward application to serial histological sections, confocal images, or micro-CT slices.

The main algorithm novelties are: (1) a new calculated area fraction variation map, which accurately illustrates the feature homogeneity in a sample; (2) a new waviness function providing insights into the convexity/concavity of pores, important for understanding the influence on cell adhesion and proliferation.

GAKTpore offers a wide range of calculated properties such as the largest sphere through a pore, porosity, local area fraction (pore dispersion/homogeneity), pore size, shape factors (circularity, waviness and aspect ratio), and pore area. The new methodology can generate large quantities of data and statistics from micrographs, facilitating improvement in process-property relationships. Advancement of their understanding can expand AI databases, improve process-property and morphology-property modelling.

GAKTpore is particularly applicable for the biomedical industry, enabling correlation of structure with cellular response. The generated bulk morphology maps allow detailed visualisation of microscopic feature variation across macroscopic length scales and significantly improving the speed and accuracy of analysis of pore sizes/shape/range/dispersion within the porous structure.

In this work, collagen scaffolds manufactured by the lyophilisation technique, previously reported homogeneous, demonstrate a heterogeneity over the bulk microstructure. With the help of the algorithm, tailoring surrogate bone strength and cell migration through modification of effective modulus and pore size/range is achievable. Furthermore, metal foam scaffolds, manufactured by the space holder technique, were found to possess agglomeration of regions denser in copper, providing insights into powder mixing efficiency.

GAKTpore possesses a large potential due to its versatility in applications and multiple novel metrics. Its computational speed will allow further implementation in the future of new image processing and feature charactarisation tools, to 3D registered data from micro-CT data sets.

## Figures and Tables

**Table 1:** Data comparing the synthetic image radius to the calculated GAKTpore radius.

Synthetic Image Max Radius (Pixels)	GAKTpore Max Radius (Pixels)	Accuracy (%)	Mean Accuracy (%)
27	26.75	99.09	99.6 ± 0.2
37	36.77	99.39	
40	39.85	99.63	
55	54.84	99.71	
63	62.84	99.75	
70	69.86	99.80	
100	99.74	99.73	
200	199.50	99.75	

**Table 2:** Accuracy of shape factors by comparing the calculated Circularity and Aspect Ratio for different shapes with values calculated from the GAKTpore algorithm.

Shape	Calculated Circularity	GAKTpore Circularity	Accuracy %	Calculated AR	GAKTpore AR	Accuracy %
Circle	1	0.999	99.9	1	1	100
Square	0.785	0.790	99.4	1	1	100
1 × 2 Rectangle	0.698	0.702	99.5	0.5	0.5	100
Equilateral Triangle	0.605	0.610	99.2	0.8660	0.8696	99.6
Hexagon	0.907	0.90981	99.7	0.8660	0.86635	99.9

**Table 3:** Table comparing the numerical waviness to the GAKTpore waviness, blue highlighted part of synthetic shape is recognised as convex and orange concave (Outer radius = 30); mean shape accuracy = 99.13%.

Synthetic Image Shape	Inner Radius	Calculated Waviness	GAKTpore Waviness	Accuracy%
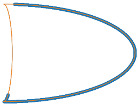	2	0.6094	0.6226	97.8
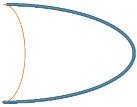	5	0.6023	0.5977	99.2
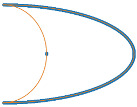	10	0.5851	0.5906	99.1
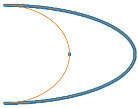	15	0.5647	0.5693	99.7
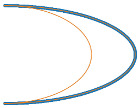	20	0.5430	0.5447	99.7
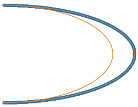	25	0.5212	0.5239	99.5
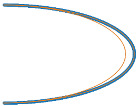	28	0.5084	0.5112	99.5

**Table 4:** The samples LSTP mean pore sizes and standard deviation compared from the GAKTpore and ImageJ measurements.

Sample	GAKTpore Mean Pore Size (µm)	σ	ImageJ Manual Mean Pore Size (µm)	σ
Porous Sintered Copper 1	5.56	1.66	5.05	2.16
Porous Sintered Copper 2	6.68	1.74	4.83	2.08
Porous Sintered Fibrous Platinum	4.68	1.52	4.40	2.12

**Table 5:** The mean shape factor (Circularity, waviness, and aspect ratio) and pore dispersion (Local area fraction) statistics with standard deviations, extracted from the sample micrographs.

Sample	Mean Circ	σ	Mean Wav	σ	Mean AR	σ	Mean Local af	σ
Porous Sintered Copper 1	0.85	0.16	0.94	0.09	0.67	0.17	0.14	0.07
Porous Sintered Copper 2	0.82	0.20	0.92	0.10	0.66	0.18	0.19	0.06
Porous Sintered Fibrous Platinum	0.83	0.16	0.94	0.08	0.61	0.17	0.12	0.06

**Table 6:** The mean pore size (LSTP), shape factor (Circularity, waviness, and aspect ratio) and pore dispersion (Local area fraction) statistics with standard deviations, extracted from the bulk sample micrographs.

Sample	Mean Pore Size (μm)	σ	Mean Circ	σ	Mean Wav	σ	Mean AR	σ	Mean Local af	σ
10 mm Porous Copper Disk	3.58	1.17	0.62	0.25	0.33	0.18	0.61	0.16	0.10	0.06
Collagen Scaffold	212.60	195.76	0.67	0.25	0.56	0.22	0.58	0.17	0.16	0.15

## Data Availability

Not applicable.

## Supplementary Materials

The GAKTpore algorithm and FFT image segmentation filter can be downloaded for use at GITHUB at https://github.com/gts4/GAKTpore (accessed on 6 March 2021). Data are available from the University of Leicester figshare Open Data Repository at https://leicester.figshare.com/ (accessed on 6 March 2021) [55].
